# Multi-Level Factors Affecting Entry into and Engagement in the HIV Continuum of Care in Iringa, Tanzania

**DOI:** 10.1371/journal.pone.0104961

**Published:** 2014-08-13

**Authors:** Erica H. Layer, Caitlin E. Kennedy, S. Wilson Beckham, Jessie K. Mbwambo, Samuel Likindikoki, Wendy W. Davis, Deanna L. Kerrigan, Heena Brahmbhatt, the LTC Tanzania Collaborative Study Team

**Affiliations:** 1 Department of Health, Behavior and Society, Johns Hopkins Bloomberg School of Public Health, Baltimore, Maryland, United States of America; 2 Department of International Health, Johns Hopkins Bloomberg School of Public Health, Baltimore, Maryland, United States of America; 3 Department of Psychiatry, Muhimbili University of Health and Allied Sciences, Dar es Salaam, Tanzania; 4 Department of Epidemiology, Johns Hopkins Bloomberg School of Public Health, Baltimore, Maryland, United States of America; 5 Department of Population, Family and Reproductive Health, Johns Hopkins Bloomberg School of Public Health, Baltimore, Maryland, United States of America; University of Washington, United States of America

## Abstract

Progression through the HIV continuum of care, from HIV testing to lifelong retention in antiretroviral therapy (ART) care and treatment programs, is critical to the success of HIV treatment and prevention efforts. However, significant losses occur at each stage of the continuum and little is known about contextual factors contributing to disengagement at these stages. This study sought to explore multi-level barriers and facilitators influencing entry into and engagement in the continuum of care in Iringa, Tanzania. We used a mixed-methods study design including facility-based assessments and interviews with providers and clients of HIV testing and treatment services; interviews, focus group discussions and observations with community-based providers and clients of HIV care and support services; and longitudinal interviews with men and women living with HIV to understand their trajectories in care. Data were analyzed using narrative analysis to identify key themes across levels and stages in the continuum of care. Participants identified multiple compounding barriers to progression through the continuum of care at the individual, facility, community and structural levels. Key barriers included the reluctance to engage in HIV services while healthy, rigid clinic policies, disrespectful treatment from service providers, stock-outs of supplies, stigma and discrimination, alternate healing systems, distance to health facilities and poverty. Social support from family, friends or support groups, home-based care providers, income generating opportunities and community mobilization activities facilitated engagement throughout the HIV continuum. Findings highlight the complex, multi-dimensional dynamics that individuals experience throughout the continuum of care and underscore the importance of a holistic and multi-level perspective to understand this process. Addressing barriers at each level is important to promoting increased engagement throughout the continuum.

## Introduction

In the past decade, the scale-up of antiretroviral therapy (ART) has led to an unprecedented number of people living with HIV (PLHIV) on treatment, resulting in decreased HIV-related morbidity, mortality and onward transmission [1], [2]. Despite these considerable gains, ART coverage remains low. In 2012, treatment coverage in low and middle income countries was estimated at 61% of all eligible individuals under the 2010 World Health Organization (WHO) HIV treatment guidelines. However, under new WHO treatment guidelines, which recommend ART initiation at ≤500 cells/mm^3^, this ART coverage represents only 34% of the people eligible in 2013 [3], [4] and considerable obstacles to universal treatment access remain.

Successful ART programs depend on the progression of PLHIV through a number of stages. This continuum of care, also referred to as a cascade of care or HIV care pathway, includes HIV testing and counseling (HTC), linkage to care (defined here as the initial engagement with the health system to receive HIV care and treatment services following an HIV diagnosis), eligibility assessment and clinical staging or CD4 testing, pre-ART care, ART initiation and lifelong ART adherence and retention in care. Considerable losses along each stage of this continuum have been well documented, especially in the pre-ART initiation [5]–[7] and retention stages [8]. However, factors contributing to suboptimal progression at each stage of the continuum are poorly understood. A systematic review of factors affecting linkages to ART in sub-Saharan Africa found that key barriers to engagement included transport costs and distance to health facilities, stigma and fear of disclosure and limited human resources [9]. MacPherson et al. reported that socio-cultural norms, support networks and limited human resources and laboratory capacity affected progression throughout the continuum in Malawi [10].

The Iringa region of Tanzania is located 500 kilometers (km) southwest of Dar es Salaam and has a population of approximately 900,000 people. The Tanzam Highway, a conduit between Dar es Salaam and Northern Zambia, cuts through Iringa, which is also home to large tea and timber plantations, leading to high rates of mobility which potentially contribute to HIV risk. Most people in Iringa are involved in small-scale agriculture [11]. A majority of the population lives more than 2 km from the nearest health facility, while over one third of rural residents live greater than 5 km away [12]. Iringa has the second highest HIV prevalence (9.1%) in the country [12]. It is estimated that only 68.6% of women and 52.7% of men in Iringa have ever been tested for HIV and received their results (26.0% and 28.2% in the past year, respectively) [12]. Thus, many individuals are unaware of their HIV serostatus and miss the opportunity for linkage to care and treatment services. Consistent with findings across sub-Saharan Africa, findings from other regions of Tanzania indicate that a substantial portion of individuals who receive a positive HIV diagnosis are not referred for subsequent care, and among those who *are* referred, many fail to register for services and clinical staging [13], [14]. Country-level estimates suggest that less than one third of those eligible for ART based on WHO 2013 guidelines are receiving it [3].

While there is considerable need to improve access to and retention in HIV services in Iringa and elsewhere, little is known about barriers or facilitators to linkages throughout the HIV continuum of care. In this study, we examined multi-level barriers and facilitators influencing entry into and engagement in the continuum of care in Iringa, Tanzania.

## Methods

Between March and November 2013 we used a mixed-methods approach to examine individual, facility and community level factors affecting linkages to HIV care and treatment.

### Facility-based data collection

At the facility level, we conducted assessments of a sample of HIV testing and treatment services, including HTC sites (n = 4), care and treatment centers (CTC) (n = 4), prevention of mother to child transmission (PMTCT) services (n = 4) and voluntary medical male circumcision (VMMC) outreach sites (n = 1). Facilities were purposively sampled throughout Iringa region to ensure diversity in urban/rural location and facility size. At each facility, we completed a structured facility checklist to gather basic operational information, a direct observation to collect information on the flow of clientele through each facility, provider-client interactions, client-client interactions, wait times, and the general ambience. In addition, convenience sampling was used to identify providers (n = 26) and HIV-infected clients (n = 75) at facilities who participated in qualitative in-depth interviews (IDIs). Facility-based data collection is summarized in Table 1.

**Table 1:** Summary of data collection across methods.

Facility-based data collection					
	Client IDIs	Provider IDIs	Direct observations	Facility checklists	
HTC (n = 4)	23	7	4	4	
CTC (n = 4)	24	8	4	4	
PMTCT (n = 4)	24	7	4	4	
VMMC (n = 1)	4	4	3	0	
**Total**	**75**	**26**	**15**	**12**	
**Community-based data collection**					
	**Client IDIs**	**Provider/key informant IDIs**	**Direct observations**	**Focus group discussions**	
Support groups (n = 5)	30	10	5	5	
Spiritual healers (n = 2)	12	4	1	0	
Traditional healers (n = 12)	0	12	2	0	
Government liaison (n = 4)	0	4	0	0	
**Total**	**42**	**30**	**8**	**5**	
**Longitudinal interview participants**					
	**Men**		**Women**		**Total**
	**ART**	**No ART**	**ART**	**No ART**	
Urban	6	7	10	8	**31**
Rural	7	4	4	2	**17**
**Total**	**13**	**11**	**14**	**10**	**48**

HTC: HIV testing and counseling; CTC: Care and treatment center; PMTCT: Prevention of mother-to-child transmission; VMMC: Voluntary medical male circumcision; IDIs: In-depth interviews; ART: Anti-retroviral therapy.

### Community-based data collection

In addition to facility-based data, we visited community-based providers of HIV care and support services, including support groups (n = 5), traditional healers (n = 12) and spiritual/religious healers (n = 2) sampled from urban and rural areas throughout Iringa region. In-depth qualitative interviews were conducted with providers (n = 52) and clients (n = 42) of these services who were conveniently sampled at these locations. IDIs were conducted with government representatives who had worked with traditional healers in the region (n = 4). We also conducted direct observations at community-based facilities. Finally, focus group discussions (FGD) were organized with existing support groups for PLHIV (n = 5) (Table 1).

### Cohort of PLHIV

To further explore the social context and dynamics of barriers and facilitators of engagement in care, we conducted longitudinal interviews with 48 PLHIV followed prospectively at three time points over the course of six months. Participants were stratified by gender, ART status and urban/rural location (Table 1).

### Data analysis

All data were collected by trained Tanzanian research assistants. Interviews and focus groups were recorded, transcribed, and translated into English. Analysis of qualitative data was conducted through a multi-stage process using a narrative and case study approach. First, each individual interview with providers and clients was summarized and re-written in brief narrative form, with particular attention to structured categories of interest related to linkages within the continuum of care. Second, for each facility, these individual narrative summaries from both client and provider interviews were brought together with data from the facility checklist and observations to develop a case summary report for that facility. In this way, the analysis brought together a variety of perspectives on individual-level, provider-level, and facility-level barriers and facilitators to engagement in HIV services for a particular setting. Similar methodology was employed for the longitudinal interviews that also sought to document the story or trajectory of each participant through the continuum of care and key factors linked to continuity or breakages in progression. Key themes were identified across these narratives and developed into a conceptual framework summarizing findings across levels and steps in the continuum of care.

### Ethics Statement

All participants provided oral informed consent prior to data collection. Written consent was not obtained because the authors felt that asking individuals to disclose their full names by providing written consent would decrease participants’ anonymity. Participants received 10,000 Tanzanian Shillings (∼USD6) at the end of each interview or FGD for their time and transport. Ethical approval, including the decision to obtain oral informed consent, was obtained from Muhimbili University of Health and Allied Sciences, the Tanzania National Institute for Medical Research and Johns Hopkins Bloomberg School of Public Health.

## Results

**Figure 1:**
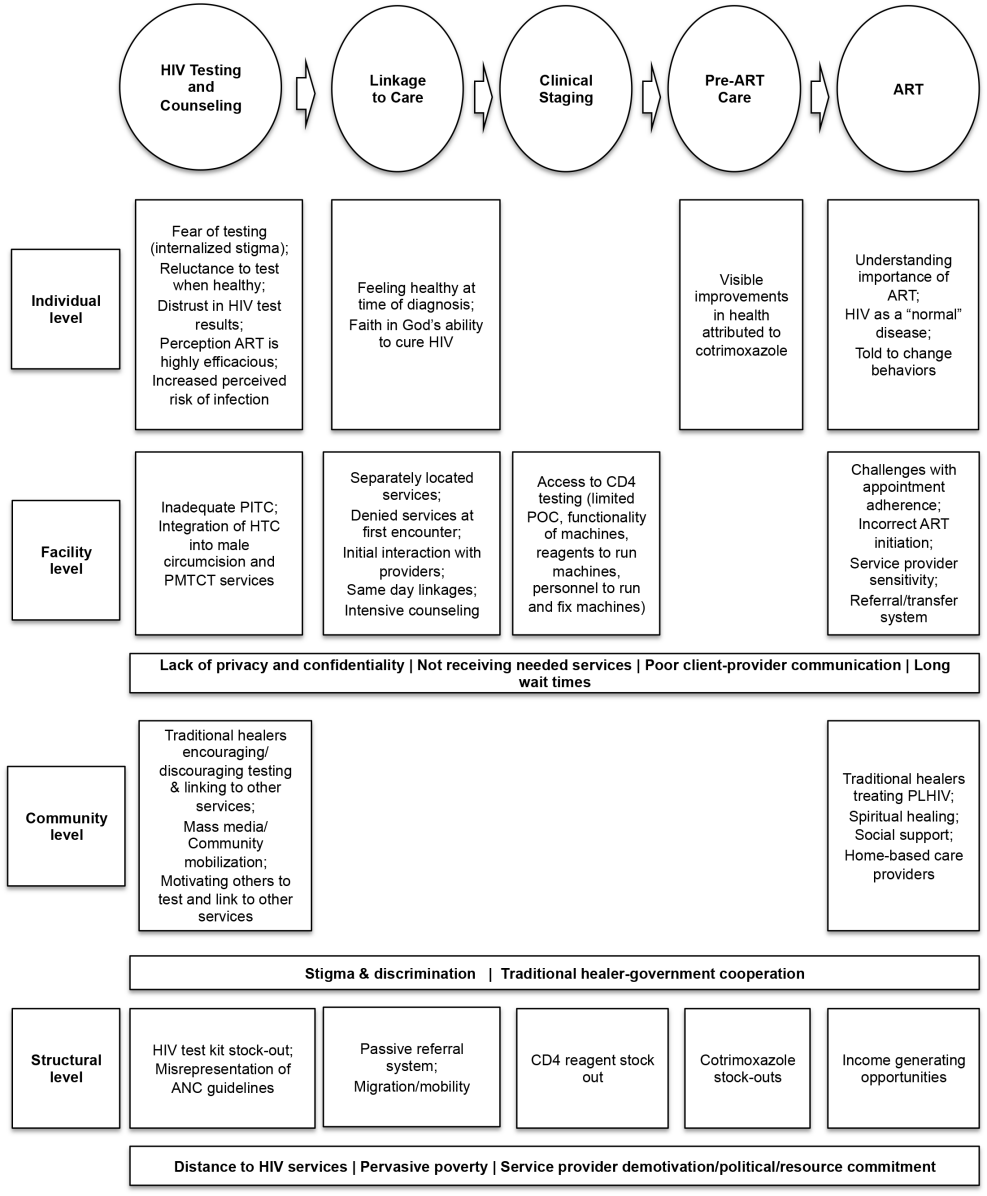
Barriers and facilitators influencing entry into and engagement in the continuum of care.

We present factors influencing engagement at each stage of the continuum of HIV care and treatment, including HTC, linkage to care, clinical staging, pre-ART care, ART and cross-cutting issues. At each stage in the HIV care continuum, we identified barriers and facilitators at the individual, facility, community, and structural levels, presented in a multi-level continuum of care framework (Figure 1). Representative quotes from key themes are summarized in Table 2. In the cohort of PLHIV, data collectors made multiple attempts to contact cohort participants for follow-up interviews. If the participant could not be reached after three separate attempts (including phone calls and visits to the participant’s home of record), the participant was designated as lost to follow up. Seven participants (3 females, 4 males) were lost to follow up during the second round of data collection and two participants (both male) were lost to follow up for the third round of data collection, for an overall retention rate of 81%.

**Table 2:** Selected themes and quotes from study participants.

LTC Stage	Theme	Quote
**1. HTC**	1.1 Fear of testing	1.1.1 “People are intimidated by the idea of testing– whether positive or negative–they are afraid. They don’t go for testing if they are not sick. They have a belief that knowing your HIV status is the end of your life. They think that you will die immediately after knowing your status.” –HTC client, M, 36
	1.2 Inadequate PITC	1.2.1 “I was treated at a dispensary at home and they failed to diagnose me. They referred me to a hospital. I was treated more than twice [without being advised to test for HIV], and then I decided by myself to check my health [get an HIV test].” –HTC client, F, 47
	1.3 HIV test kitstock-out	1.3.1 “We are losing so many clients. It’s very disgusting for someone who leaves his house knowing he is going to the hospital for services. [He thinks]“I am going to get [HTC] services.” And then when he gets there, he finds there are no services. It’s not only here; this is the problem for all places in this district. I think it’s also in all places in Tanzania … So that’s a big problem. We face some difficulties in delivering HCT services.” –HTC provider, F, 55
	1.4 Denying pregnantwomen ANC/PMTCTservices until partnerattends	1.4.1 “When women go to the [ANC] clinic, they are asked to bring their husbands. They don’t get other services until they bring their husbands. So the husbands are motivated because they tell themselves that their partner won’t get the services unless they bring them to the clinic and they can’t stay without those services. That’s how they get tested [for HIV].” -PMTCTProvider, F, 41; 1.4.2 “I already knew that when you go to the ANCclinic for the first time, you must go with a man - your partner. But he was not present; whenever I made a phone call to him he would say, “I will come” or “I will make arrangements.” Time was passing and even when I came for the first time, the nurse sent me back … The nurse told me, “Go to advise your husband [to come with you to the clinic] so that we can help you.” -PMTCT client, F, 31
	1.5 Visiting traditionalhealers	1.5.1 “I took my wife to a traditional healer. I thought she was bewitched. I thought she was bewitched because she was being treated [at the hospital] but did not heal. What will he tell you? He will tell you that he has the medicine and that the patient has been bewitched. He cannot tell you that she has HIV. They want her to suffer and most of them are liars … There isn’t one [who would advise a client to go to a hospital].” –Cohort participant, M, 30
	1.6 Belief that ARTis efficacious	1.6.1 “I came here [for HTC] voluntarily. I decided to check on my HIV status because a lot of people who get tested get [ART] services and are doing well. I decided to come here for testing so that I will start taking [ART] medications before my health deteriorates. If they find that I am HIV infected, I know I will comply well with the treatment regimen and there will be few side effects … Your health will be at stake if you wait for a fever before you get tested.” –HTC client, F, 30
	1.7 PLHIV testimonials	1.7.1 “We help people to realize that HIV is not such a scary disease … There are a lot of people who are going for testing right now due to our influence. Some come here for advice. They go for HIV testing and are initiated on ART. They are doing well now because of us. They were so afraid of HIV testing before the group was established. I decided to disclose my status in public to save others. Some people who have symptoms come to me for advice. I tell them about everything that they have to do so that they are started on medications.” –Support group member, F, 34
**2. Linkage to** **care**	2.1 Feeling healthyat diagnosis	2.1.1 “I saw that my body was good and I didn’t have any problem. I was not sick so I decided to stay strong like that without following up on anything.” –Cohort participant, F, 28
	2.2 Separately locatedservices	2.2.1 “[It’s challenging when a client] has tested in centers that do not have other services. You give the client a referral letter to go to another place. When he reaches a certain place [CTC] and starts hesitating, he will stop. But if he tested here, he would be escorted to the CTC by the VCT service provider.” –CTC provider, F, 38
	2.3 Challenges duringinitial linkage	2.3.1 “When I reached [the doctor’s room at the CTC] they told me that I should go back to the reception. When I saw that man [from reception] he was so strict. He said ‘I told you to go there [to the doctor].’ But I said, ‘They told me that I should come back here [to reception].’ But he insisted that I should go up. Then I went back upstairs again. When I reached there, they asked what is wrong with me. I told them, ‘I was sent to bring you this note [referral letter from HTC]. I totally don’t understand the process in this place.’ Then they said since the doctor is not around I should leave and come back on Tuesday.” –HTC client, M, 33; 2.3.2 “I would expect that when you come here [to the CTC] for the first time, providers should explain what you are supposed to do. But here they are so rude and say, “We have already told you to do this and this” when they haven’t told you anything. So it happensthat you don’t understand what to do because they fail to explain, and if you make a little mistake then they start to yell at you, and so it happens that youstart answering them rudely.” -HTC client, M, 36
**3. Clinical** **staging and** **CD4 testing**	3.1 Ineffective systemsfor CD4 testing	3.1.1 “It was troublesome; we used to wake up at 6 am to get [CD4] testing. When you reach there, you find a long line of people and the machine takes only 50 patients, so when you reach 50 it was finished. The others [who did not get tested] had to leave; I had to go there for about a week. I managed to get tested in the second week … You have to wake up about 4 or 5 in the morning so that you can be early; when you are later than that you get turned away.” –CTC client, F, 45
**4. Pre-ART** **care**	4.1 Cotrimoxazolestock-outs	4.1.1 “Frankly speaking there is not enough [cotrimoxazole]. Most of the time they are disturbing us so much. And for some people who are starting [pre-ART care], when they are coming for the first month, they are told to go and buy them [cotrimoxazole], and in the next month they are told to go and buy them, and in the next month they are told to go and buy them. Now it reaches a point when he sees that he would be better off to go and buy themrather than coming here [to the CTC]; he is wasting his time.” -Client, M, 45
**5. ART** **initiation,** **adherence and** **retention**	5.1 HIV as a “normal”disease	5.1.1 “During the beginning when we were getting this [CTC] service there used to be very few people. So I felt very bad when I was told that I had theinfection … later on I felt normal as they [CTC providers] continued giving me this medication [ART], so as the number of people kept on increasing, I kept on encouraging myself that it is better if I continue using the medication. Initially I felt so weak, I felt like my fellows were somehow stigmatizing me. But after the number of people kept on increasing thenI felt like it is just a normal thing and since then I have beenfeeling good.” -Client, M, 47
	5.2 Disrespectfultreatment by serviceproviders	5.2.1 “When I reached there [CTC] they said, ‘How many are late?’ We raised our hands. They said, ‘Every day we tell you to come early. When is your time?’ One of us raised our hand and said, ‘At 8 AM.’ The provider said, ‘Why are you coming at 9 and we have so much work to do? …Today you will get service at 12.’ And surely we were seen at 12. They left, I don’tknow where, maybe to drink tea, until we got tired, that is when they came back and gave us services.” -Cohort participant, F, 42; 5.2.2 “I was on the waiting bench. That’s where they weigh us before we enter into the doctor’sroom. That’s when a nurse said, ‘You are wearing tight pants and the way you are seated is seductive and you have even applied eye liner. Who are you trying to attract? You just want to hurt others [i.e. infect others].’ That was so painful … I stopped attending those services [at the regional hospital]because of the statements used in there. I just stayed home because I had already lost hope because of the statements used by some nurses over there. There were very good services until one nurse spoke to me in a very badway that made me feel worthless, maybe because of the way I am. So I felt really sad. My heart doesn’t feel like going back there because I feel sad every time I see her.” –CTC client, F, 31
	5.3 Spiritual healing	5.3.1 “PLHIV can go for the [ART] medications, but we know that it is prayers that sets them free from their problems, not medication. I can say that the medications have their own position but it’s very minor, prayers arethe main deal.” – Spiritual healer, F, 38
	5.4 Support groups	5.4.1 “Disclosing your status makes you strong in such a way that you can’t be shaken by any enemy who will try to speak against your status. First and foremost I have my freedom, I don’t have bitterness. I have peace of mind wherever I go; it’s because of this group.”-Support group member, F, 34
	5.5 Home basedcare providers	5.5.1 “It is easy [to follow up with clients who miss appointments] currently because of these HBCs. We just look where [the patient] comes from and then we just use the HBC from there. [The HBC] tries to follow [the patient], sometimes someone may die and we don’t have the information. So [the HBC] will give us the information … If [the patient] just decided not to come, then [the HBC] will also tell us, but currently, by using the HBCs, a majority [of patients] have returned back to the service.” -Provider, F, 43
**6. Cross-cutting**	6.1 Not receivingservices	6.1.1 “The ones who are missing CTC services are so many because of thedistance. Someone comes, yet he is being told that maybe [cotrimoxazole] isnot in stock, you see. And most of them are told to go and buy. Then he comes in the next month then they are telling him, ‘You have not had your CD4 checked so we cannot change the medications for you [i.e. initiate ART], so you are supposed to go and buy [cotrimoxazole] again.’ So that situation is what leads to that [dropping out of services]. First, he is lookingat the distance and he is wasting his time. On the day that he comes, he hasto prepare the whole day for not working. So when he prepares that day andthen he sees that he doesn’t get that service, he sees that it is not a productivework going there [to the CTC].” -Client, M, 45
	6.2 Stigma anddiscrimination	6.2.1 “I felt that I was discriminated against by my relatives. My relativesdid not even contribute a cent to help me nor did they escort me [to the hospital]. And it is not that my relatives do not have the means, they are people who are able to. I was really discriminated [against]. Even my neighbors, my in-laws would even ban their children from coming to my home. They would say, ‘You better not eat anything there.’ One time, my sister-in-law got sick when she was pregnant, but she said, ‘I will try, better I hire someone [else] to take care of me. Because my sister-in-law has AIDS,she might infect me.’ Therefore I felt bad.” –Client, F, 39
	6.3 Service providerburnout	6.3.1 “We are losing hope because we do this hard job with no motivationof any kind. The work is very hard. You just receive people’s problems andyou are supposed to help them, but there is nothing that you are going to gain, so it really breaks our hearts … The work is very hard but the government cannot help us … There are other times that our fellows [otherservice providers] have no plans to help patients who are admitted in the ward who need that service. He might be sick in bed, but he is not attended … If there were incentives then someone would work wholeheartedly.” –CTC provider, F, 55

### HIV testing and counseling

HTC is available in Iringa through a mix of client-initiated and provider-initiated services. Client-initiated voluntary counseling and testing services are offered through both static and mobile services. Provider-initiated testing and counseling (PITC) should be routinely practiced in all health facilities. In addition, PITC services are offered routinely with VMMC and antenatal care (ANC) services.

Study participants described a perception that HIV is characterized by severe illness and that infected individuals would be visibly sick. Participants described a strong reluctance to test while healthy and said that individuals often distrust positive test results if they are not sick and therefore often seek multiple HIV tests to confirm positive results. Fear of the stress of an HIV-positive test result and internalized stigma were additional individual-level barriers to HTC, especially among asymptomatic individuals (Table 2, quote 1.1).

A majority of our participants received HTC only after being sick for extended periods of time. Medical care was sought at multiple health facilities for recurring illnesses but HTC was rarely recommended during these visits. Despite the government policy of PITC, this service was not routinely offered to participants in our study and providers missed critical cases among these clients (Table 2, quote 1.2.1).

Persistent stock-outs of HIV test kits, which were common throughout Iringa region for the duration of this study, prevented access to HTC services. Service providers noted that they routinely turned clients away, while clients explained that the stock-out had caused many people to give up on HIV testing completely (Table 2, quote 1.3.1).

In addition, women in our study were often denied antenatal care (ANC) services unless they brought their male partners to the first ANC visit for couples HIV testing. During a direct observation of a PMTCT facility, a data collector noted a poster on the wall that stated (in Swahili), “It is required for a pregnant woman to come with her husband/partner on her first [ANC] visit. You will not be served without following this.” Multiple women in our study reported being denied ANC services because they were not accompanied by a male partner, and participants shared stories of friends who delayed ANC services or avoided them altogether (Table 2, quote 1.4.1 and 1.4.2). While Tanzania national guidelines do not support this practice, they do encourage increased male participation and recommend that ANC providers promote “couple/partner HIV/STI testing and counseling for all young women” [15]. Service providers interviewed for this study explained that they required women to bring their partners in order to increase male involvement, which appears to be a misrepresentation of national guidelines, with the unintended negative consequence of discouraging single women from seeking care.

Traditional healers offered an alternative to the formal health system and some individuals attended these services after multiple failed attempts to determine the cause of their illness. Clients of traditional healers were rarely advised to test for HIV and noted they were always told that they were bewitched and in need of traditional medicine, preventing further engagement with the health system (Table 2, quote 1.5.1).

Several factors facilitated HTC, including the perception that ART is highly efficacious, often as a result of witnessing health improvements when a friend or relative initiated ART; increased perceived risk of infection resulting from an AIDS-related death of a spouse or family member; or having multiple sexual partners (Table 2, quote 1.6.1). Mass media promoting HTC, mobile HIV testing services, community mobilization and PLHIV testimonials were additional facilitating factors (Table 2, quote 1.7.1).

### Access to and linkage to care

Following HTC, all additional HIV services in Iringa are provided at CTCs. The linkage to care phase includes the process of a newly diagnosed HIV-infected individual successfully progressing from an HTC facility to a CTC for assessment of ART eligibility.

One key facilitator prompting linkage to care was severe illness at the time of HIV diagnosis. These clients expressed relief in determining the cause of their illness and were happy to immediately link to a CTC in order to initiate treatment. In contrast, participants widely acknowledged that asymptomatic individuals were much less likely to link to a CTC because they either did not believe the HIV test results or did not see the point in receiving care (Table 2, quote 2.1.1). Several clients of spiritual healing services also delayed linking to care because they had faith that God would either cure them or stop disease progression.

Co-located HTC and CTC services facilitated linkages to care. Participants agreed that linking a person to a CTC on the same day increased that person’s chance of continuing to receive HIV services, especially when a service provider personally escorted the client. However, many HTC facilities were stand-alone, so clients were given a referral card and told to travel to the nearest CTC on their own which was noted as a key point at which individuals drop out of services (Table 2, quote 2.2.1). In addition, providers had no way to follow up with patients to ensure successful linkage to treatment and care services because no active tracking systems existed in the facilities we assessed.

Clients often encountered challenges during their initial visit and were told to leave and return on another day due to restricted opening hours, limited capacity for enrollment and shortages of providers (Table 2, quote 2.3.1). Some participants also expressed frustration at the way they were treated by CTC providers during their initial visit (Table 2, quote 2.3.2).

### Clinical Staging and CD4 testing

After successfully linking to HIV care and treatment services, clients undergo clinical and/or laboratory staging to determine whether they are eligible for either pre-ART or ART care. In Iringa, this process most commonly involved a medical evaluation and CD4 testing. According to national guidelines, ART initiation is recommended for individuals with WHO stage 3 and 4 clinical criteria, regardless of CD4 cell count, and for adults and adolescents with a CD4 count <350 cells/mm3, regardless of clinical symptoms [15].

While government guidelines allow for ART initiation in individuals based on clinical evaluation, all participants in our study reported being required to receive CD4 testing before ART initiation. This process was a major challenge for most CTC clients in this study. From the results of the facility checklist, we found that of the four CTC facilities visited, only one had a working CD4 machine, while two had machines that were either broken or lacked reagents. Clients were required to get a referral letter from their original CTC, travel long distances, were often turned away and test results were commonly lost, forcing clients to repeat the process multiple times (Table 2, quote 3.1.1). Because of these inconveniences, participants said that many PLHIV lose hope and drop out of HIV services at this stage.

### Pre-ART care

Following staging, PLHIV who are not yet eligible for ART engage in pre-ART care services until they are eligible to initiate ART. This stage in the continuum of care should include regular clinical assessments for ART eligibility and consistent HIV care. According to Tanzanian guidelines, individuals in this stage of the continuum of care should receive cotrimoxazole prophylaxis, a combination of antibiotics used to treat a range of opportunistic infections associated with HIV [15]. Clients are expected to return to the CTC monthly for monitoring and to receive cotrimoxazole, free of charge, and should receive CD4 testing every six months until eligible for ART initiation.

Many ART-ineligible clients who were enrolled in pre-ART care viewed receiving cotrimoxazole as the main benefit of attending monthly appointments. In fact, several clients attributed dramatic improvements in their health to this medication. Unfortunately, chronic stock-outs were common; six out of 11 facilities reported cotrimoxazole stock-outs at the time of the facility checklist and study participants were often told to purchase the medication from a private pharmacy. Participants listed this as a significant factor for drop out of pre-ART care services (Table 2, quote 4.1.1).

### ART initiation, adherence and retention

The next stage in the continuum of care is ART initiation. Current Tanzanian national guidelines recommend ART for individuals with CD4 counts of ≤350 cells/mm3 or those with severe or advanced clinical disease. After initiating ART, PLHIV are counseled to adhere to daily ART regimens and should return for regular clinic appointments for assessment, counseling and medication refills.

Many ART clients reported high levels of ART adherence due to health improvements experienced after ART initiation. Additionally, clients were motivated to continue attending CTC services because seeing other PLHIV made them feel that they were “not alone” and helped them to see HIV as a “normal” disease (Table 2, quote 5.1.1).

There were potential provider-level factors that acted as barriers to ART initiation and adherence. Several cohort participants reported that they were told by their doctors to discontinue ART when they became healthy. Participants also noted that they were not initiated on ART, even after their clinical symptoms indicated ART initiation or when their CD4 count was well below 200. However, these situations were reported by only a few study participants and were not supported by observations or interviews with providers, so the generalizability of these findings is not known.

One of the most significant barriers to retention in CTC services was disrespectful and abusive treatment by service providers. Almost all participants in this study encountered negative experiences where they were shouted at, “scolded” or “punished” by one or more providers. Often, negative interactions occurred when a client disobeyed rules set by providers, most commonly arriving late or missing an appointment (Table 2, quote 5.2.1). When clients returned to the CTC on a day other than their assigned clinic day, they were often either denied services completely or forced to wait until the end of the day as punishment or “correction” for their behavior. Harsh and disrespectful treatment was the most common reason for CTC clients to disengage from care (Table 2, quote 5.2.2).

Visiting traditional and spiritual healers were common alternate pathways to care. While most traditional healers said that they were unable to treat HIV, several noted that they routinely diagnosed, treated and cured PLHIV. In addition, *all* spiritual healers expressed faith in God’s ability to cure HIV and encouraged their clients to trust in God’s healing powers, and one spiritual healer explained that prayers helped PLHIV more than ART (Table 2, quote 5.3.1). A majority of alternate healers said that while they did not discourage clients from attending HIV services, they generally did not actively encourage attendance at HIV services, and several participants knew of people who were encouraged to stop taking ART while participating in spiritual healing services. Most clients of traditional and spiritual healers in this study attended alternate healing services in parallel with CTC services. These individuals were aware of the importance of ART for their survival, but had hope that they might be cured through alternate healing systems, so continued to attend both services. Clients of spiritual healers also discussed the support they received from other PLHIV during healing services as a reason for continued attendance.

Support groups for PLHIV were identified throughout Iringa region. These groups were generally initiated and managed by PLHIV in the community and members attended regular meetings. Each group visited in this study also had an economic strengthening component through income generating activities or savings programs. Members of PLHIV support groups benefitted in many ways which facilitated their involvement in CTC services, including through social support and income generating opportunities. Most group members said they were no longer “embarrassed” of being HIV-positive and did not feel ashamed to attend CTC services because they knew that they were not alone (Table 2, quote 5.4.1). Clients of HIV services who were not members of support groups expressed their desire for more opportunities for support and income generating activities but were not aware of organized groups in the area.

Home based care providers (HBCs), community volunteers who serve as a link between the health system and community by visiting PLHIV in their homes and following up after missed appointments, were present in two CTC facilities in this study. In these facilities, HBCs were mentioned as a very effective system for following up with patients who did not attend their CTC visits since they lived and worked at the village level. Service providers at the only CTC facility with HBCs said that they rarely lost clients to follow-up due to the strong support provided by HBCs (Table 2, quote 5.5.1).

### Cross-cutting themes

In addition to the themes presented above which correspond to a specific stage within the continuum of care, several cross-cutting themes were identified which influenced engagement in HIV services at all stages.

Participants described frustration after traveling long distances for care only to be turned away if they arrived outside of clinic hours, if providers were too busy to serve them, if there were stock-outs of HIV test kits and cotrimoxazole or when they encountered challenges accessing CD4 testing services (Table 2, quote 6.1.1). Poor communication between providers and clients limited clients’ abilities to fully understand their situation, which led to misunderstanding important concepts such as what CD4 levels mean, the difference between cotrimoxazole and ART, and the importance of lifelong ART adherence. Finally, long wait times and severe crowding, especially at CTC facilities, caused frustration and led some PLHIV to disengage from care.

Stigma, discrimination and internalized stigma were very common themes throughout this study which impeded individuals’ ability to access and participate in HIV services. Participants commonly reported that people avoided HTC due to fear of discrimination they would face if others discovered their HIV status, and many CTCs had waiting areas or queues that were outside, making the patients visible to all other hospital attendees or even people passing by. Some CTC clients traveled long distances to avoid seeing people that they knew at CTC facilities in their communities, while others discussed the “humiliation” they were forced to endure every time they attended CTC services. Many clients of HIV services also shared experiences of being discriminated against in their communities (Table 2, quote 6.2.1). Because of the real and perceived social stigma, many participants had not disclosed their HIV status to anyone outside of their immediate family, and many had not disclosed to their spouses due to fear of violence or abandonment.

Service providers discussed challenges they faced which ultimately led to burn out and lower quality care, including severe shortages of staff, lack of incentives and inadequate training. Doctors were often assigned to multiple departments while providers mentioned that they turned clients away from HTC or CTC services if they came towards the end of the day, since they were too exhausted to see more clients (Table 2, quote 6.3.1).

### Individual trajectories

While the results presented above highlight specific factors which affect an individual’s decision to engage in HIV services, these single barriers and facilitators do not capture the complexity of participant experiences, which often involve multiple competing priorities and challenges. To illustrate this complexity, we present three case studies from participants in the longitudinal cohort to contextualize the themes within a person’s life trajectory. All names are pseudonyms.

#### Emmanuel: Disengagement following poor quality care

In 2011, Emmanuel’s wife was pregnant. She told him that it was mandatory for men to accompany their wives to ANC services for HIV testing; if he did not attend, his wife would not receive services. Emmanuel hesitantly agreed to accompany his wife. At this visit, he was diagnosed with HIV even though he felt healthy and had no visible signs of illness.

His wife encouraged him to pursue further HIV care services, so he immediately travelled to the nearest CTC which was more than two hours away via public transportation. However, he became frustrated with the poor quality of care and long distances and disengaged from HIV services after only four visits.

Emmanuel’s decision to disengage from care was a culmination of several factors, including inadequate services, being told repeatedly to return on a different day, and the cost associated with each trip. During his visits, he noted that he was not provided with any education or medication. He explained, “I gave them my [CTC] card and they would tell me to come back the next month. I would go the next month, again they would write for me [to come back the next month], without getting any medication.” Similarly, he tried four times to receive his CD4 test results but was denied every time and told to return on another day. Emmanuel explained that he could not justify wasting his time and money on CTC visits when the quality of services was so low and he was in good health, so he disengaged from care and never returned.

#### Linda: Internalized stigma

In 2005, Linda was diagnosed with HIV after being chronically ill for several years. Before her HIV diagnosis, Linda had frequent fevers, chest pain and tuberculosis. Even after completing tuberculosis treatment, she continued to experience recurrent illness but had never been advised to test for HIV by health providers. After ruling out all other possible problems, Linda decided to receive HTC.

After receiving her HIV-positive diagnosis, Linda was shocked and contemplated suicide. She recalled, “I wished to take ten tablets at once so that I would die. That’s what I was thinking.” However, several service providers supported her and convinced her to accept her diagnosis and engage in HIV care and treatment services.

Linda did not disclose her HIV status to her husband or her children for one year due to fear of abandonment and discrimination. She believed that people perceived HIV to be a disease brought about by casual sex, and thought that PLHIV were viewed as “hooligans” or “prostitutes.” Linda found this particularly challenging because she was an older woman and felt that she was being harshly judged by people in her community.

Linda’s decision to receive treatment was challenging and complex. When she arrived for services, extremely long lines, poor ventilation, and waiting for long periods in public where others could identify her as HIV-positive made her feel humiliated. Despite these challenges, Linda continued to engage in HIV services, which is due in large part to her participation in a support group for PLHIV. The group helped her to cope with the challenges she faced while waiting for CTC services and made her understand the importance of ART adherence for her survival.

#### Furaha: Verbal abuse from service providers

Furaha was diagnosed with HIV in 2007 after suffering from recurrent illnesses for more than one year. She was relieved when she received her HIV diagnosis because she finally discovered what was bothering her and felt happy that she could get relief. She said that her health improved dramatically after ART initiation and she attended CTC services regularly for five years. In 2012 however, she disengaged from care due to missing one CTC appointment because she was working away from home. When she returned on another date, she was scolded and yelled at by the providers who refused to give her ART. She tried to return several weeks later but was still denied services as a punishment for missing one appointment and did not want to continue to face service providers who treated her so poorly.

During the period that she was disengaged from care, Furaha purchased cotrimoxazole from the pharmacy and “borrowed” ART from her friends who were engaged in CTC services. She expressed concern and anxiety about not being able to adhere to ART and said, “I have to go back because I don’t want to die. I have to take the medications to stay alive …I pray that they accept me without scolding”.

During the course of this study, Furaha was successfully able to re-engage in care. The doctor “warned” her not to repeat missing appointments, but agreed to allow her to resume ART, but only after repeating three weeks of ART training.

## Discussion

Understanding factors which motivate and prevent PLHIV from engaging in and adhering to care at each step along the continuum is critical to successful HIV treatment and prevention efforts. Our findings are consistent with previous studies assessing barriers and facilitators throughout various stages of the HIV care continuum [9], [10], [16]–[19]. While many of the barriers presented here have been looked at independently as factors associated with negative care outcomes, few if any studies have looked holistically at all of these multiple levels and types of factors influencing the full continuum of care in a given setting. This study highlights the complex interplay of these factors and the need to provide comprehensive solutions which address the multiple challenges to providing HIV treatment and care services.

An individual’s health was a strong influencing factor in progression through the continuum of care. Those who were visibly sick and had ruled out other causes of disease were most likely to seek HTC services, accept their diagnosis and immediately link to care and treatment. Further, these individuals were also the most likely to see dramatic improvements in their health after initiating cotrimoxazole and/or ART, and therefore viewed these medications as important for sustained health. In contrast, healthy participants in our study expressed reluctance to receive HTC and were more likely to delay linking to care or disengage from care and treatment services, which is consistent with other findings throughout sub-Saharan Africa [20]–[23]. When faced with additional barriers to care such as long distances, high transport costs, stigma and risk of verbal abuse from providers, these individuals often chose not to engage in care because the perceived need for medical care was outweighed by multiple barriers and competing priorities. As treatment guidelines evolve to recommend ART initiation at higher CD4 counts, more people will initiate ART before a noticeable decline in health. Identifying these individuals and ensuring successful progression through the continuum of care is critical, but will be challenging. Fully implementing the policy of universal and routine PITC services in all health facilities to normalize HTC, and behavior change communication strategies to promote earlier testing and engagement in care, could change the current social norms around HIV testing and promote earlier uptake of HIV services.

Persistent stock-out of supplies, including HIV test kits, CD4 reagents and cotrimoxazole were common throughout Iringa region during this study. In addition to causing frustration and demotivation among service providers and clients, these stock-outs decreased trust in the health system, promoted disengagement from care and led to poor health outcomes. HTC providers noted that they routinely turned clients away during frequent HIV test kit stock-outs and believed that these people would give up and not return at a later date. In addition, lack of timely CD4 counts due to broken machines or missing reagents led to an inability to determine ART eligibility and has been shown delay ART initiation [10], [24], [25]. Provision of free cotrimoxazole, which was described by our study participants as the most important element of pre-ART care, has been shown to double retention in pre-ART care services [26]. Unfortunately, this benefit was undermined by chronic cotrimoxazole stock-outs which led to deep dissatisfaction and reduced clients’ perceived need to attend monthly visits. Participants in our study who did not believe they would receive required services were less likely to invest in the time, money and effort needed to attend visits to health facilities. Stock-outs of HIV-related medication and supplies have been documented in other African settings [27]–[30] and these findings highlight the need to strengthen supply chain management systems. In addition, point of care CD4 testing, which provides immediate results for use in patient care, could eliminate many of the logistical and operational barriers to CD4 testing noted in this study [31]–[33].

Provider attitudes and treatment of clients were significant barriers to retention in care and the main reason for disengagement from CTC services among clients of ART care. Many study participants endured verbal abuse and disrespectful treatment by providers at CTC facilities because accepting this mistreatment was the only way to receive ART. However, several clients disengaged from care when they could no longer handle being degraded, ridiculed and punished, even though they knew their health would suffer as a consequence. These findings point to a clear need to improve provider-client interactions as a means of reducing disengagement from care. Physical and verbal abuse against patients has been reported in a variety of health settings throughout sub-Saharan Africa [34]–[37]. In a study in South Africa, Jewkes et al. described providers’ abusive treatment as “a complex interplay of concerns including organizational issues, professional insecurities, perceived need to assert ‘control’ over the environment and sanctioning of the use of coercive and punitive measures to do so, and an underpinning ideology of patient inferiority” [38]. They explained that violence was allowed to become commonplace due to lack of accountability and limited action taken by managers against service providers who abuse patients, which appears consistent with our findings. Service providers in our study discussed burnout and demotivation as a result of staff shortages, unrealistic workloads and lack of supervision and training. Health-system level changes to increase human resources, provide incentives, ensure adequate support systems and provide ongoing training and supervision are needed to increase service provider motivation and improve client-provider interactions.

Consistent with other studies, our findings suggest that HIV-related stigma and discrimination are key barriers to engagement in HIV services throughout the continuum, leading to suboptimal levels of HTC [39], disclosure [40], retention in care [41] and ART adherence [42], [43]. Negative health outcomes resulting from HIV-related stigma have been well documented [44]–[48] and our findings highlight the need for stigma-reduction strategies to accompany HIV prevention and treatment efforts. While effective interventions to reduce community-level stigma and damaging social norms are not well-defined [49], there is general consensus that that four basic approaches are effective in reducing stigmatizing attitudes among individuals and groups, including information, skills-building, counselling and PLHIV testimonials [50], [51].

Community-level approaches to HIV service delivery could be one strategy to reduce barriers at multiple levels identified in our study. A growing body of evidence suggests that novel strategies to bring HIV services to the community level through task shifting, such as home-based HIV testing [52]–[55] and home-based ART initiation [56] and delivery [57]–[61] are effective, feasible and acceptable [62]. In addition, positive side effects of home-based ART delivery programs include increased community-level social support and decreased discrimination [61], [63], and clients of these services reported saving time and money due to reduced clinic visits [64]. These novel community-based strategies would require significant political commitment and operational research to tailor programs to the local context, but may have the potential to strengthen all stages of the HIV care continuum by improving identification of HIV-infected individuals, simplifying linkages to care, improving retention in ART care programs, and reducing structural-level barriers such as distance, cost and stigma and discrimination.

Finally, leveraging and expanding services and opportunities identified in this study which facilitate engagement in care, including PLHIV support groups, income generating opportunities, HBC providers and government engagement with alternate healing systems, could strengthen linkages to care and help to reduce the impact of additional barriers to engagement and retention in care in this setting.

Strengths of this study include the diversity of respondents and data collection methods which provided multiple perspectives on factors affecting the continuum of care in Iringa. In addition, the longitudinal nature of the qualitative cohort allowed us to gain an in-depth understanding of the complexity of PLHIV’s experiences as they moved through the continuum of care. At the facility level, our interviews with clients of HIV services and service providers, direct observations and facility checklists allowed us to triangulate findings. In addition, interviews and focus group discussions with members of support groups, traditional healers and spiritual healers add a unique dimension and understanding to community-level influence throughout the continuum of care.

Despite these strengths, the study has several limitations. First, HIV test kit stock-outs limited our ability to interview newly diagnosed clients, especially those who chose not to link to further care and treatment services, which could have provided valuable insight into barriers at this stage. Another limitation was our inability to recruit HIV-infected clients of traditional healers. Traditional healers universally noted that PLHIV do not disclose their status, and they therefore were not aware of any HIV-positive clients. While we would have liked to understand the perspective of this group, we were able to capture experiences from longitudinal cohort participants who had visited traditional healers themselves or knew of people who had.

## Conclusion

This study presents a multi-level framework for understanding barriers and facilitators to linkages to care in Iringa, Tanzania. Our findings highlight the complex, multi-dimensional dynamics that individuals experience throughout the continuum of care and underscore the importance of taking a holistic and multi-level perspective to understand this process. Interventions to address single barriers identified in this study are insufficient; our findings illustrate how multiple barriers interact and influence decisions about engagement in care. Addressing barriers at multiple levels is needed to promote increased engagement and retention in care.
